# Cognitive impairment with Type 2 Diabetes Mellitus among community-dwelling older adults in Chile: Prevalence, risk factors and cognitive characteristics

**DOI:** 10.3389/fnhum.2022.1070611

**Published:** 2023-01-19

**Authors:** Agnieszka Bozanic, Pablo Toro, Sebastián Bello-Lepe, Javier Hurtado-Oliva, Christian Beyle, Catalina Valdés, Francesc Formiga

**Affiliations:** ^1^Facultad de Educación y Ciencias Sociales, Universidad Andres Bello, Viña del Mar, Chile; ^2^Department of Psychiatry, Faculty of Medicine, Pontificia Universidad Católica de Chile, Santiago, Chile; ^3^Advanced Center for Chronic Diseases, Pontificia Universidad Católica de Chile, Santiago, Chile; ^4^School of Speech Therapy, Valparaiso University, Viña del Mar, Chile; ^5^Department of Otorhinolaryngology, Head and Neck Surgery, University Medical Center Groningen, University of Groningen, Groningen, Netherlands; ^6^Psychology Department, Universidad Católica de Temuco, Temuco, Chile; ^7^Department of Health, Universidad de Los Lagos, Osorno, Chile; ^8^Geriatric Unit, Internal Medicine, Hospital Universitari de Bellvitge, IDIBELL, L’Hospitalet de Llobregat, Barcelona, Spain

**Keywords:** Type 2 Diabetes Mellitus, dementia, Mild Cognitive Impairment, prevalence, cross-sectional study

## Abstract

**Introduction:**

The aim of this study is to determine prevalence and risk factors of Cognitive Impairment (CI) and its association with Type 2 Diabetes Mellitus (T2DM) in subjects aged 65 years and above. Additionally, we attempt to provide a cognitive profile for T2DM group.

**Methodology:**

A cross-sectional analytical study to assess CI was carried out. We evaluated a sample of community-dwelling residents from Chile. All participants underwent a general interview, lifestyle questionnaires and a comprehensive neuropsychological battery. Regression analyses were performed to evaluate risk of CI with T2DM and influencing factors. Results between groups in the different domains of the neuropsychological assessment were compared by Student’s *t*-tests and MANOVA.

**Results:**

Among all 358 subjects, overall T2DM prevalence were 17.3%. The prevalence of CI was higher in T2DM group compared to the healthy participants (30.7%, *p* < 0.001). The risk of CI was 2.8 times higher in older people with T2DM compared to older people without the diagnosis. Multiple regression analysis, adjusted for age and gender, demonstrated that age, education, presence of dyslipidemia, and T2DM duration were the predictor variables significantly associated with CI. T2DM group performed worse on global cognitive performance, attention, language, verbal memory, visual memory, visual constructional ability, and executive function. After adjusting for significant covariates from multiple regression analysis, a relationship between “cognition” and T2DM is still observed. Amnesic multi-domain impairment was the specific cognitive identified pattern for T2DM group.

**Conclusion:**

The present study confirms the high prevalence of CI with T2DM among Chilean older adults in a community-based population. T2DM is significantly associated with a higher risk of CI, and age, education, presence of dyslipidemia, and duration of T2DM are risk factors. T2DM patients with CI are impaired in multiple cognitive domains, even after adjusting covariables, resulting in an amnesic multi-domain cognitive profile.

## Introduction

Type 2 Diabetes Mellitus (T2DM) is a metabolic disease associated with modifiable and non-modifiable risk factors (International Diabetes Federation, 2015). Regarding non-modifiable factors, there is clear evidence that certain ethnic groups are at increased risk for developing T2DM (Centers for Disease Control and Prevention, 2017). Latin-Americans have a higher prevalence of T2DM than Caucasian people (Centers for Disease Control and Prevention, 2017), have a higher risk, almost twice, of developing T2DM (American Diabetes Association, 2022) and show high levels of disease burden and complications such as higher rates of kidney failure, diabetes-related vision loss and blindness (Centers for Disease Control and Prevention, 2022). In Chile, the prevalence of T2DM reaches 12.3% of the general population and 30.6% of people 65 years or over (Miniesterio de Salud de Chile, 2017), placing it in second place of South America (International Diabetes Federation, 2015) and in 5th place among all OECD countries (Organisation for Economic Cooperation and Development, 2011).

Public awareness of T2DM is mainly restricted to medical complications such as microvascular, macrovascular, and non-vascular complications (American Diabetes Association, 2022). However, there are critical cognitive aspects that need increased attention. Multiple studies have demonstrated the existence of an association between T2DM and Cognitive Impairment (CI), which are presented in a continuum ranging (Bozanic et al., 2019) from subtle cognitive decreases associated with T2DM (Koekkoek et al., 2015), cognitive decline (Biessels and Despa, 2018), Mild Cognitive Impairment (MCI) (Roberts et al., 2014) to dementia (Cheng et al., 2012). The CI with T2DM has been described as a decrease in cognitive performance in specific domains such as verbal and visual memory, attention and concentration, processing speed, and executive function (Palta et al., 2014). Despite research showing that T2DM causes brain-related cognitive dysfunction, a clinically useful pattern associated with T2DM have not yet established.

A recent systematic review and meta-analysis of observational studies have reported a general combined prevalence of CI with T2DM of 45% (You et al., 2021), with significant differences between countries. Specifically, in China 13.5% (Gao et al., 2016), India 33.7% (Khullar et al., 2016), France 28.8% (Verny et al., 2015), Spain 26.1% (Rodríguez-Sánchez et al., 2016), and Mexico 27.4% (Arjona-Villicaña et al., 2014).

Studies of community-dwelling individuals suggested that the probability of developing CI is 1.39–2.9 times higher in people with T2DM than without the disease (Cheng et al., 2012; Roberts et al., 2014). There is clear evidence that attributed the increased risk of CI with T2DM to poor lifestyle, age, hyperglycemia, hypercholesterolemia (Sharma et al., 2020), T2DM duration (Bozanic et al., 2021), and symptomatic and asymptomatic cerebrovascular disease (Araki and Ito, 2002), among others.

To our knowledge, this is the first study about the prevalence of CI with T2DM and its influencing factors in Chilean population. On the other hand, few Hispanic studies analyze cognitive characteristics using a comprehensive neuropsychological test battery to study CI among older adults with T2DM. Therefore, the present study aimed to estimate the prevalence of CI and its associated risk factors in the Chilean older adult population with T2DM. A secondary aim was to examine differences in cognitive performance between older Chilean adults with T2DM and healthy controls to provide a cognitive profile.

## Methodology

An observational, descriptive, comparative, and cross-sectional population study was conducted between October 2017 to September 2019. The sample was taken from the DIABDEM (DIABetes and DEMentia) project, whose methodology has already been published elsewhere (Bozanic et al., 2019). The DIABDEM project aimed to determine the prevalence of CI and its risk factors in people aged 65 or over with T2DM in Spain and Chile for people 65 or over who lived community-dwelling.

The reference population was Chilean inhabitants in 2017 (17.574.003 inhabitants total), from which 11.4% (2.003.256) were aged 65 or older (Instituto Nacional de Estadísticas, 2018). The sample was taken from 6 different Chilean cities. Recruitment was carried out by invitation to: (1) older adults who participate in Senior Social Clubs contacted by listing independently of active participation in the organization, and (2) older adults attending primary care centers contacted by listing independently of periodic visits. Each participant underwent an in-person interview by trained interviewers administered. The standard assessment included medical history, lifestyle questionnaires, and a comprehensive neuropsychological battery.

## Participants

A random sample from the DIABDEM project comprised 358 subjects representing the adult population of Chile age 65 and older. Exclusion criteria were as follows: (1) have another nationality than Chilean, (2) live in another country, (3) visual, auditory, or motor impairments that precluded neuropsychological assessments; (4) history of dementia; (5) active psychiatric symptomatology that interfered with the ability to complete neuropsychological assessments.

The study was approved by an independent Ethics Committee of Scientific Research from Pontificia Universidad Católica de Chile (ID 170516002 project) and all participants were provided with written informed consent, according to the recommendations of the Declaration of Helsinki.

## Variables

### Sociodemographic and clinical information

Sociodemographic data (age, gender, marital status) and years of education were collected. We classified each patient’s level of education as primary education (1 to 6 years of education), secondary education (7 to 12 years of education), and higher education (more than 13 years). Also, the participants were asked whether they lived alone or were accompanied, whether they presented subjective memory complaints, and the time since the diagnosis of T2DM was made (0–5 years, ≥5 years). We classified participants as suffering from hypertension, dyslipidemia, kidney failure, or heart disease if there is a previous diagnosis or if they received drug treatment to control these diseases. Obesity was classified as normal weight, overweight or obese according to the body mass index (BMI) (Cervi et al., 2005).

We calculated comorbidity with the Charlson Comorbidity Index (Núñez et al., 2004). Lifestyle risk factors were defined as: (a) smoking: regular consumption of tobacco in the last year, and (b) a sedentary lifestyle: assessed by the Spanish version of the Rapid Assessment of Physical Activity (Topolski et al., 2006), (c) alcohol consumption: self-reported consumption of at least one unit per day, (d) low adherence to the Mediterranean diet: assessed by the Spanish version of Mediterranean Diet Adherence Screener (Martínez-González et al., 2012). Finally, functional impairment in instrumental and basic activities of daily living was measured with the Spanish version of the Brody Instrumental Activities of Daily Living Scale (IADL) and the Barthel Index for Activities of Daily Living (ADL) (Olazarán et al., 2005).

### Diabetes

Diabetes was assessed by self-report, considered a reliable and valid indicator of a diabetic status (American Diabetes Association, 2022). We also assessed diabetes when a person oral hypoglycemic agent and/or insulin and must have been diagnosed with T2DM in the 3rd or later decades of their life (American Diabetes Association, 2022).

### Neuropsychological assessment

The present study employed the comprehensive clinical neuropsychological battery that has previously been reported (Bozanic et al., 2019). For the assessment of global cognitive performance, the Chilean version of the Mini-Mental State Examination (MMSE-Ch) was used (Quiroga et al., 2004). Attention was assessed by the Spanish version of Trail Making Test (TMT-A) (Tamayo et al., 2012), and Forward Digit Span of the Spanish version of Weschler Adult Intelligence Scale (WAIS- IV) (De la Guia et al., 2012). We assessed language using the Spanish version of Boston Naming Test (BNT) (Olabarrieta-Landa et al., 2005). Verbal memory was assessed by the Spanish version of words recall Free and Cue Selective Reminding Test (FCRST-w) (Olabarrieta-Landa et al., 2005). Visual memory was assessed using Deferred Copy of the Spanish version of Rey Osterrieth Complex Figure (ROCF) (Palomo et al., 2013). The visuo-constructional ability was measured by the Spanish version of Rey Osterrieth Complex Figure (ROCF) (Palomo et al., 2013). Finally, executive function was assessed by the Spanish version of Trail Making Test (TMT-B) (Tamayo et al., 2012), Backward Digit Span, Symbol Digit and Similarities of the Spanish version of Weschler Adult Intelligence Scale (WAIS-IV) (De la Guia et al., 2012; Tamayo et al., 2012), a Spanish version of semantic (animals) and Spanish version of phonological fluency (P) (Peña-Casanova et al., 2009).

### Cognitive impairment

Subjects were classified as having CI by two different methods:

Method 1: CI was defined if subjects met 1.5 standard deviations performance on the general cognitive performance test below published norms according to age and education level.

Method 2: CI was defined if subjects met the following criteria (Albert et al., 2011):

– 1.5 standard deviations performance on the general cognitive performance test below published norms according to age and education level.

– Functional impairment on instrumental or basic activities of daily living.

– Subjective memory complaint reported by self-report.

## Statistical analyses

Data were analyzed using the Statistical Package for the Social Sciences version 20.0 (SPSS Inc., Chicago, IL, USA). A value of *p* < 0.05 was considered statistically significant. The chi-squared and Student’s *t*-test were used for discrete and continuous variables, respectively. Logistic regression was conducted to examine the association between T2DM and CI. The multiple logistic regression model included variables that had a *p* < 0.25 in the simple logistic regression analysis (age, gender, education, presence of dyslipidemia, and duration of T2DM). A MANOVA analysis of “cognition” was performed to determine the impact of T2DM diagnosis on cognitive functioning after adjusting with covariates found on multiple logistic regression. The variable “cognition” is composed of cognitive performance tests scores of Mini Mental State Examination Chile (MMSE-Ch), Free and Cue Selective Reminding Test (FCSRT)- words; Trail Making Test A (TMT-A), Trail Making Test B (TMT-B), Weschler Adult Intelligence Scale (WAIS-IV), Rey Osterreich Complex Figure (ROCF), and Boston Naming Test (BNT). Interactions effects were explored within covariates to analyze the relationship between factors on cognitive performing. F, Cohens, IC 90%, and *p*-values were provided.

## Results

### Demographic and clinical characteristics

The average age of the 358 participants was 68.9 ± 4.8 years, 12.0 ± 4.0 years of education, and 78% of the participants were women. The prevalence of prior T2DM diagnosis was 17.3%, leading a group of 62 participants in the T2DM group and 296 participants in the non-T2DM group. In participants with T2DM, 54,5% were women, they were older (70.8 ± 4.46 years vs. 68.5 ± 4.79 years *p* < 0.001) and had fewer years of education (10.4 ± 4.53 years vs. 12.4 ± 3.84 years, *p* < 0.001) in comparison with those without T2DM, respectively (Table 1).

**TABLE 1:** Sociodemographic characteristics of the participants comparing people without Type 2 Diabetes Mellitus and patients with Type 2 Diabetes Mellitus.

	Non-T2DM *n* (%)	T2DM *n* (%)	Chi-Sq or *t*-test *P*-value
Total	296 (82.7)	62 (17.3)	–
Age (years)	68.5 ± 4.8	70.8 ± 4.5	<0.001**
Gender			<0.001**
Male	49 (16.6)	22 (35.5)	
Female	247 (83.4)	40 (64.5)	
Education (years)	12.4 ± 3.8	10.4 ± 4.5	<0.001**
Marital status			0.423
Widow	44 (14.9)	7 (11.2)	
Single	18 (6.1)	2 (3.2)	
Married	212 (71.6)	45 (72.6)	
Separated	22 (7.4)	8 (12.9)	
Live accompanied			0.800
No	28 (10.7)	4 (7.8)	
With a partner	223 (82.3)	47 (92.2)	

Percentages, averages, and their standard deviation are presented. **Significant differences.

As shown in Table 2, T2DM subjects had a greater presence of subjective memory complains (71% vs. 53.4%, *p* = 0.016), dyslipidemia (54.8% vs. 23.3%, *p* = < 0.001), kidney 250 failure (6.4% vs. 1.7%, *p* = 0.052), comorbidity (21% vs. 4.1%, *p* = < 0.001), CI (38.8% vs. 13.85%, *p* = < 0.001), low adherence to the Mediterranean Diet (90.3% vs. 62.8%, *p* = < 0.001), and were more likely to be sedentary (9.7% vs. 20.6%, *p* = 0.045) than the non-T2DM subjects. It was not possible to collect data of heart diseases and obesity.

**TABLE 2:** Clinical characteristics, comorbidity, and Lifestyle risk factors, of the participants comparing people without Type 2 Diabetes Mellitus and participants with Type 2 Diabetes Mellitus.

	Non-diabetic *n* (%)	Diabetic *n* (%)	*P*-value
Subjective memory complains	158 (53.4)	44 (71)	0.016**
Hypertension	201 (68)	49 (79)	0.113
Dyslipidemia	69 (23.3)	34 (54.8)	<0.001**
Kidney failure	5 (1.7)	4 (6.4)	0.052**
Smoking	70 (19)	17 (26.2)	0.203
Alcohol consumption	12 (3.1)	5 (8.1)	0.066
Sedentary lifestyle	61 (20.6)	6 (9.7)	0.045**
Mediterranean Diet Adherence	110 (37.2)	6 (9.7)	<0.001**
Functional impairment IADL	1 (0.34)	0 (0)	0.827
Functional impairment ADL	9 (3.04)	5 (8.06)	0.083
Cognitive impairment Method 1	41 (13.85)	19 (30.65)	<0.001**
Cognitive impairment Method 2	119 (40.2)	33 (53.2)	0.081

IADL, instrumental activities of daily living; BADL, basic activities of daily living. Percentages are presented. **Significant differences.

### T2DM as risk factor of cognitive impairment

Participants with T2DM had significantly higher prevalence of CI compared with non-T2DM subjects (see Table 3). Method 1 has shown that the risk of CI is 2.8 times higher in older people with T2DM compared to older people without this diagnosis. Method 2 did not show significant differences.

**TABLE 3:** Cognitive impairment of the participants comparing people without Type 2 Diabetes Mellitus and participants with Type 2 Diabetes Mellitus.

	OR (95% IC)	*P*-value
**Cognitive impairment method 1**		
No	Ref	
Yes	2.8 (1.78–4.40)	<0.0001**
**Cognitive impairment method 2**		
No	Ref	
Yes	1.7 (0.9–2.9)	0.056

Data are presented as Odds Risk (OR) and their respective confidence intervals (95% CI).

**Significant differences.

### Risk factors for CI in T2DM participants

Binary logistic regression analysis was performed with age, gender, years of education, marital status, living status, subjective memory complaints, comorbidities, hypertension, dyslipidemia, kidney failure, duration of T2DM, age when T2DM was diagnosed, smoking, alcohol consumption, adherence to the Mediterranean diet, and sedentary lifestyle, as independent variables, and the incidence of CI (method 1) as the dependent variable. From this analysis, significant differences between T2DM participants with CI and those with normal cognitive function were found in most clinical indicators, but not in sociodemographic and lifestyle risk factors, except in adherence to the Mediterranean diet (OR = 0.19, IC 95% = [0.07, 0.55], *p* = < 0.01) (see Table 4).

**TABLE 4:** Single logistic regression analysis of associations between Type 2 Diabetes Mellitus with Cognitive Impairment participants and sociodemographic, clinical, and lifestyle variables.

Variables	Crude OR (95% CI)	Wald statistic	*P*-value
**Sociodemographic**			
Age (years)	1.06 (0.99–1.13)	3.43	0.640
**Gender**			
Male	1		
Female	0.46 (0.23–0.92)	4.79	0.030**
Education (years)	0.86 (0.79–0.93)	13.29	0.010**
**Marital status**			
Married	1		
Single	1.09 (0.73–1.63)	0.17	0.680
**Living status**			
Accompanied	1		
Alone	1 (0.99–1,01)	0.01	0.940
**Clinical**			
**Subjective memory complaints**			
No	1		
Yes	2.85 (1.36–5.98)	7.67	0.010**
**Comorbidities**			
No	1		
Yes	2.10 (1.38–3.19)	12.09	0.010**
**Hypertension**			
No	1		
Yes	2.43 (1.04–5.64)	4.25	0.040**
**Dyslipidemia**			
No	1		
Yes	4.22 (2.19–8.15)	18.41	0.010**
**Kidney Failure**			
No	1		
Yes	2.15 (0.43–10.68)	0.87	0.350
**Duration T2DM**			
> 5 years	1		
< 5 years	6.36 (4.19–9.65)	75.43	<0.001**
**Treatment**			
Insulin	1		
Metformin	0.99 (0.98–1.01)	0.06	0.810
**Lifestyle**			
**Smocking**			
No	1		
Yes	1.21 (0.71–2.07)	0.49	0.490
**Alcohol consumption**			
No	1		
Yes	1.23 (0.41–3.66)	0.14	0.710
**Sedentary lifestyle**			
No	1		
Yes	1.16 (0.83–1.63)	0.74	0.390
**Mediterranean Diet Adherence**			
No	1		
Yes	0.19 (0.07–0.53)	9.78	<0.001**

Data are presented as Odds Risk (OR) and their respective confidence intervals (95% CI).

**Significant differences.

The multivariate logistic regression model was statistically significant, χ^2^ = 19.91, *p* = < 0.01, explaining 63.5% of the variance in the presence of CI among T2DM participants (Nagelkerke *R*^2^). Age, education, presence of dyslipidemia, and T2DM duration appeared as independent predictor of CI with T2DM (see Table 5).

**TABLE 5:** Multivariate logistic regression analysis of associations between Type 2 Diabetes Mellitus with Cognitive Impairment participants and sociodemographic, clinical, and lifestyle variables.

		OR (CI 95%)	*P*-value
Model 1	Duration T2DM	6.36 (4.19–9.65)	0.000**
	Education	0.86 (0.77–0.96)	0.006**
Model 2	Duration T2DM	6.51 (4.22–10.04)	0.000**
	Education	0.88 (0.79–0.99)	0.028**
Model 3	Dyslipidemia Duration T2DM	0.375 (0.14–0.99)	0.049**
		6.25 (4.04–9.67)	0.000**
	Age	0.86 (0.75–0.98)	0.025**
	Education	0.82 (0.72–0.94)	0.004**
Model 4	Dyslipidemia	3.27 (0.11–0.84)	0.021**
	Duration T2DM	7.56 (4.52–12.65)	0.000**

Data are presented as Odds Risk (OR) and their respective confidence intervals (95% CI).

**Significant differences.

### Cognitive characteristics in T2DM participants

According to cognitive performance assessments, participants with T2DM had significantly worse cognitive performance indicators in all domains tested (see Table 6). A MANOVA analysis revealed a significant effect of all the covariates (age, gender, education, presence of Dyslipidemia and T2DM duration) on cognition. Interaction effects within covariates were studied, founding not significant for Age*Presence of Dyslipidemia, Gender*Education, Gender*T2DM duration and Presence of Dyslipidemia*T2DM duration (see Table 7).

**TABLE 6:** Cognitive performance assessments scores of the participants comparing people without and with Type 2 Diabetes Mellitus.

	Non-T2DM (± DE)	T2DM (± DE)	IC 95%	*P*-value
MMSE-Ch	28.6 (1.64)	27.7 (1.79)	28.2–28.6	<0.001**
**FCSRT-w**				
Free recall	29.5 (7.9)	24.1 (8.7)	27.7–29.5	<0.001**
Cued recall	44.8 (5.6)	40.9 (9.7)	43.4–44.8	0.044**
Total recall	14.2 (3.5)	13.5 (3.4)	13.7–14.5	0.004**
TMT-A	76.5 (35.7)	98.1 (62.0)	75.4–84.7	0.034**
TMT-B	140.9 (64.6)	203.9 (58.5)	143.9–158.9	<0.001**
BNT	38.7 (16.4)	24.3 (8.4)	34.5–37.9	<0.001**
**ROCF**				
Copy	29.9 (8.5)	22.9 (12.2)	27.6–29.7	<0.001**
Copy time	222.2 (92.5)	248.8 (113.5)	216.7–236.8	0.048**
Deferred Copy	15.3 (8.1)	8.2 (6.5)	13.2–15.09	< 0.001**
**WAIS IV**				
Forward Digit Span	5.3 (2.1)	6.7 (2.3)	5.3–5.8	<0.001**
Backward Digit Span	4.4 (1.6)	4.4 (1.7)	4.2–4.5	0.946
Similarities	22.2 (9.4)	15.1 (7.4)	19.9–21.9	<0.0001**
Symbol Digit	30.8 (12.7)	28.9 (16.3)	29.0–31.9	0.364
Semantic fluency (animals)	17.6 (7.3)	16.7 (7.6)	16.6–18.2	0.375
Phonologic fluency (P)	13.5 (4.4)	11.7 (4.8)	12.7–13.7	0.003**

MMSE-Ch, Mini Mental State Examination Chile; FCSRT- words, Free and Cue Selective Reminding Test; TMT-A, Trail Making Test A; TMT-B, Trail Making Test B; WAIS-IV, Weschler Adult Intelligence Scale; ROCF, Rey Osterreich Complex Figure; BNT, Boston Naming Test. TMT-A, TMT-B, Copy time ROCF in seconds. Averages and their standard deviation are presented.

**Significant differences.

**TABLE 7:** MANOVA of “cognition.”

	*F* (1,273)	Eta2 (partial)	Cohen	IC 90%	*P*-value
Age	9.94	0.37	0.58	0.27–0.41	<0.001**
Gender	4.36	0.20	0.25	0.10–0.24	<0.001**
Education	19.81	0.53	1.15	0.46–0.58	<0.001**
Presence of Dyslipidemia	6.34	0.27	0.37	0.16–0.31	<0.001**
T2DM duration	5.16	0.23	0.30	0.12–0.27	<0.001**
Age*Gender	1.72	0.13	0.10	0.03–1.00	0.047**
Age*Education	2.07	0.44	0.12	0.35–1.00	0.012**
Age* Presence of Dyslipidemia	1.28	0.19	0.07	0.09–1.00	0.218
Age* T2DM duration	1.78	0.18	0.10	0.08–1.00	0.037**
Gender*Education	1.14	0.52	0.07	0.43–1.00	0.324
Gender * Presence of Dyslipidemia	2.13	0.26	0.12	0.15–1.00	0.009**
Gender * T2DM duration	1.53	0.22	0.09	0.11–1.00	0.094
Education*Presence of Dyslipidemia	2.23	0.21	0.13	0.10–1.00	0.006**
Education*T2DM duration	2.91	0.21	0.17	0.10–1.00	<0.001**
Presence of Dyslipidemia * T2DM duration	0.65	0.18	0.04	0.08–1.00	0.834

“Cognition”: cognitive performance tests scores of Mini Mental State Examination Chile (MMSE-Ch). FCSRT- words, Free and Cue Selective Reminding Test; TMT-A, Trail Making Test A; TMT-B, Trail Making Test B; WAIS-IV, Weschler Adult Intelligence Scale; ROCF, Rey Osterreich Complex Figure; BNT, Boston Naming Test. Data are presented as Odds Risk (OR) and their respective confidence intervals (95% CI).

**Significant differences.

## Discussion

Our results indicated that the prevalence of CI with T2DM was 30.6%. Multiple studies demonstrated different rates of prevalence of CI with T2DM across ethnicity (Noble et al., 2012). T2DM with CI was more prevalent among African Americans and Hispanics than Caucasians (11.4 vs. 4.9%; *p* = 0.06) (Albert et al., 2011). This finding was even slightly higher than studies conducted on Hispanic populations in Spain and Mexico (Arjona-Villicaña et al., 2014; Rodríguez-Sánchez et al., 2016). Chile has a distinctive epidemiological profile. So, ethnicity, demographics, nutritional, and physical activity changes may be playing a crucial role in increasing the prevalence of metabolic risk factors and accelerating the incidence of diseases like T2DM and, possibly, comorbid CI.

According to method 1, people with T2DM are 2.8 times as likely to show a risk associated with CI, which is consistent with previous cross-sectional and longitudinal studies that demonstrated epidemiological evidence suggesting that T2DM is associated with CI at 2.5 times greater risk than subjects without the disease (Cheng et al., 2012). We found no significant differences between the groups using method 2, diagnostic criteria recommended by the National Institute on Aging and Alzheimer’s Association workgroup (Cervi et al., 2005). We theorize that this disparity could be associated with the high functional performance of the evaluated sample. In addition, only a low number of participants showed reduced IADL or ADL.

Our study proven a significant association between CI and T2DM with age and years of education as protective factors, and presence of Dyslipidemia, and duration of T2DM as risk factors. Several studies showed that age is a primary risk factor for cognitive decline. Further, CI has been shown to be correlated with brain structure and function (Naninck et al., 2017) and a decreased cognitive performance on tasks for memory and information-processing speed was associated with increased age in T2DM people (Ruis et al., 2009). Education levels were one of the strongest predictors of maintained cognitive function (Albert et al., 1995; Rowe and Kahn, 1997). Some studies commented that education plays a more significant role than age in cognitive test performance (de Azeredo Passos et al., 2015). Of note, people with T2DM and low education showed poor cognitive performance (Teixeira et al., 2020; Kowall and Rathmann, 2022).

Even though it is unclear if hyperlipidemia or hypercholesterolemia contributes to cognitive decline or having a risk of developing Alzheimer Disease in T2DM subjects, it is well known that hypercholesterolemia and hypertriglyceridemia are associated with an increased risk for Alzheimer Disease (Appleton et al., 2017). Thus, further clinical studies on the impact of hyperlipidemia on the risks of cognitive impairment in T2DM subjects are needed. An unhealthy lifestyle is considered the major contributor to T2DM, causing a progressive deterioration of mental health. Finally, T2DM duration is another crucial contributor to CI. Cognitive decline is more prominent when the duration of Diabetes is more than 5 years (Bozanic et al., 2021). Besides, a long T2DM diagnosis significantly influences the conversion to dementia in T2DM with CI patients (Albai et al., 2019).

These findings supported our prediction that T2DM negatively impacts cognitive functioning. The T2DM group consistently performed worse on all measures, showing a decline in global cognitive performance but also in specific cognitive fields such as attention, language, verbal memory, visual memory, visual constructional ability, and executive function, as previous studies showed (Palta et al., 2014; Teixeira et al., 2020). Even after adjusting by cofounders as age, gender, education, presence of dyslipidemia, and duration of T2DM, T2DM significantly impacted cognitive function negatively. The influence of T2DM on cognition interacts with other dimensions. For instance, older individuals with T2DM and lower education tend to perform lower cognitive testing. Thus, age and education are factors to consider when individuals with T2DM perform cognitive assessments. It was suggested that pathophysiologic changes related with advancing age and low education, in addition to T2DM, has synergistic effects resulting in higher CI.

These functions are particularly relevant because they involve behaviors such as problem- solving, judgment, and changing habits that be crucial in prescribing complex tasks (Nici and Hom, 2019). Despite current recommendations for an annual cognitive assessment as a good practice in treating subjects with T2DM (Srikanth et al., 2020), a structured approach for evaluating cognitive impairment associated with T2DM is not currently part of daily practice. Moreover, this geriatric syndrome is not yet considered one of the main complications of the disease. There is also no standard cognitive assessment battery to assess cognitive impairment associated with T2DM specifically. However, a comprehensive neuropsychological assessment has been strongly proposed, including measuring different cognitive domains beyond an essential cognitive screening (American Diabetes Association, 2022).

Our data suggest a specific pattern for impaired function in the affected cognitive domains: amnesic multi-domain impairment. “Diabetes-related dementia” presents a unique pattern of pathologies in the cognitive domain (Hanyu, 2019). Neuroimaging has shown a more significant impairment in memory and executive function than that experienced in people with Alzheimer’s disease (Hanyu, 2019). Despite pathological differences, people with dementia-related to T2DM are often screened for cognitive status using the same brief cognitive assessment tests as people with Alzheimer’s disease, such as the Mini-Mental State Examination (MMSE), which is not suitable. It is suggested to apply the MoCA test, which has proven to be a brief, simple and effective tool to detect cognitive impairment in T2DM population (Mordenfeld et al., 2020).

Characterization of cognitive performance patterns, and their associated risk factors, could provide a basis for the early screening and intervention of CI in Older adults. Detection of CI and an accurate neurocognitive profile could help clinicians to give recommendations to maintain an adequate self-management of T2DM and mitigate poor clinical outcomes for patients and their families, keeping lining the incidence of all kinds of diabetes-related complications. A high degree of vigilance for cognitive deficits should be maintained in this population, particularly in those with additional risk factors for CI (Hanyu, 2019). Therefore, unifying the diagnostic criteria for CI with T2DM in the community-dwelling population was highly urgent.

There are several strengths of this study. First, due to the high heterogeneity of the population over 65, it is common for geriatric research to focus research and conclusions on a specific geriatric subpopulation. We do not claim the representativeness of the entire older population.

However, our results could be extrapolated to the community-dwelling older adults population and relatively robust users of outpatient primary health care settings. Second, our study utilized a comprehensive neuropsychological evaluation that allowed us to observe the performance in detail of different cognitive domains and thus propose a specific cognitive profile. However, it presents some limitations as well. First, a causal relationship between cognition and T2DM could not be concluded because of the cross-sectional nature of the observation. Second, considering the lack of laboratory data and diagnostic classifications based on participant responses, the prevalence of CI underlying diseases might be underestimated. Heart diseases and obesity could be independent factors of cognitive impairment, so it will be important to collect this data on the future. Third, we did not focus the analyses on the relationship between the different subtypes of cognitive impairment and cognitive function. Finally, longitudinal data from larger samples are needed to verify the above hypothesis.

## Conclusion

Our ongoing research has found a higher prevalence of CI with T2DM among Chilean older adults in a community-based population aged 65. T2DM is significantly associated with a higher risk of CI, and education, presence of dyslipidemia, and duration of T2DM are risk factors in CI with T2DM people. T2DM patients with CI are impaired in multiple cognitive fields, such as general mental status, attention, language, verbal memory, visual memory, constructional ability, and executive function, even adjusting by cofounders, providing an amnesic multi-domain cognitive profile.

These findings will help policymakers estimate the potential burden of T2DM in healthcare settings, specifically among middle- and low-income Latin-American countries. Our data demonstrate that even a brief cognition evaluation is essential to assess the impact of T2DM on the mental health of this population, so these types of preventive strategies have been taken in order to avoid worsening cognition functions in these high-risk individuals.

## Data availability statement

The raw data supporting the conclusions of this article will be made available by the authors, without undue reservation.

## Ethics statement

The studies involving human participants were reviewed and approved by Ethics Committee of Scientific Research from Pontificia Universidad Católica de Chile (ID 170516002 project). The patients/participants provided their written informed consent to participate in this study.

## Author contributions

AB contributed to the conception or design of the work. AB, PT, and SB-L contributed to interpretation of data for the work and drafting the work. SB-L contributed with data analysis. SB-L, CB, JH-O, and CV contributed to data collection. PT, SB-L, CB, JH-O, CV, and FF revising it critically for important intellectual content and final approval of the version to be published. All authors contributed to the article and approved the submitted version.
